# Therapeutic alliance in early schizophrenia spectrum disorders: a cross-sectional study

**DOI:** 10.1186/1744-859X-12-14

**Published:** 2013-05-09

**Authors:** Ragnhild Johansen, Valentina C Iversen, Ingrid Melle, Knut A Hestad

**Affiliations:** 1 Forensic Department Brøset, Centre for Research and Education in Forensic Psychiatry, St. Olav’s Hospital, Trondheim University Hospital, P. Box 3008 Lade, Trondheim, 7441, Norway; 2 Department of Psychology, The Norwegian University of Science and Technology, Trondheim, 7491, Norway; 3 Division of Mental Health Care, Innlandet Hospital Trust, Brumunddal, 2380, Norway; 4 St. Olav’s Hospital, Trondheim University Hospital, Trondheim, 7006, Norway; 5 Department of Neuroscience, Faculty of Medicine, The Norwegian University of Science and Technology, Trondheim, 7491, Norway; 6 Department of Psychiatry, Institute of Clinical Medicine, University of Oslo, Oslo, 0318, Norway; 7 K.G. Jebsen Centre for Psychosis Research, Oslo University Hospital, Oslo, 0407, Norway

**Keywords:** Schizophrenia, Alliance, Symptom, Psychosocial treatment, Agreement

## Abstract

**Background:**

The therapeutic alliance is related to better course and outcome of treatment in schizophrenia. This study explores predictors and characteristics of the therapeutic alliance in recent-onset schizophrenia spectrum disorders including the agreement between patient and therapist alliance ratings.

**Methods:**

Forty-two patients were assessed with demographic, neurocognitive, and clinical measures including the Positive and Negative Syndrome Scale (PANSS). The therapeutic alliance was measured with the Working Alliance Inventory - Short Form (WAI-S).

**Results:**

Patient WAI-S total scores were predicted by age and PANSS excitative symptoms. Therapist WAI-S total scores were predicted by PANSS insight. Patient and therapist WAI-S total scores were moderately associated. Neurocognition was not associated with working alliance.

**Conclusion:**

Working alliance is associated with specific demographic and symptom characteristics in patients with recent-onset schizophrenia spectrum disorders. There is moderate agreement between patients and therapists on the total quality of their working alliance. Findings highlight aspects that may increase therapists’ specificity in the use of alliance-enhancing strategies.

## Background

Schizophrenia spectrum disorders present with disabling psychiatric symptoms. Most patients also have a broad range of difficulties in social and occupational functioning and extensive, long-term need for health care services. Inadequately treated patients risk a poorer prognosis and a poorer quality of life [1]. The first 2 to 5 years of illness are considered a critical period in the development of psychotic disorders. It is during this period that adequate treatment may substantially impact the course and outcome of illness [2,3]. A recent meta-analysis [4] however indicates a drop-out rate of 13% for the psychosocial treatment of schizophrenia spectrum disorders. It is thus important to uncover factors that have impact on the quality and outcome of treatments. Low engagement with treatment, as assessed using the Service Engagement Scale [5], is associated with several patient-related factors, including the therapeutic alliance [6], clinical symptoms (Positive and Negative Syndrome Scale (PANSS) positive and excitative symptoms), and neurocognitive measures (conceptualization) [7].

The therapeutic alliance is widely recognized as a common therapeutic factor, which is critical for treatment success across different treatments and patient groups [8]. Authors use different terms to describe this alliance; however, the terms most often used are therapeutic, helping, or working alliance, the latter referring to Bordin’s [9] formulation of a therapeutic relationship defined by the level of agreement on the tasks and goals of therapy as well as the development of a personal bond between patient and therapist.

Several studies show that the therapeutic alliance also is important in schizophrenia and related to important aspects of treatment such as better compliance with medication, lower drop-out rates, fewer rehospitalizations, improved symptom levels, and better outcomes [10-13]. Interest has over time turned towards more in-depth examination of factors that can influence the quality of the therapeutic alliance, including the degree of shared opinion between patient and therapist regarding the quality of the alliance [14-18]. Problems relating to the experience of a positive emotional bond between patient and therapist or the lack of agreement on the tasks or goals of therapy have been defined by previous authors as ruptures to the therapeutic alliance and shown to be related to treatment outcome [19]. In the treatment of patients with psychotic illness, some reason that the therapeutic alliance is particularly vulnerable to such ruptures, as delusional understanding of context or poor insight is more frequent in these patient groups [20].

Initially, the idea of a functional working alliance could appear at odds with the perception of patients with schizophrenia suffering from reality distortion or having lack of insight into their disorder. Results from schizophrenia spectrum studies have here been somewhat conflicting, in that some have found that the level of psychotic symptoms is associated with lower patient ratings of the alliance, whereas others have not [15,21,22]. Higher levels of insight seem, with one exception only [23], to be consistently associated with higher patient ratings of the therapeutic alliance [15,17,21] and also with therapist-rated alliance in one study [21]. In addition, therapists seem to report higher ratings of their alliance with patients who are presenting better social abilities [13,14,21] than those who do not. While neurocognitive factors have been shown to influence the level of service engagement, there are limited reports of their relation to working alliance. A small study of 24 patients with chronic schizophrenia spectrum disorders found that poorer verbal memory was associated with better patient ratings of working alliance, while better therapist ratings were associated with better visuo-spatial reasoning. The authors discussed the possibility that patients and therapists are affected in different manners by the patient’s neurocognitive abilities [24].

Results from studies investigating patients’ and therapists’ agreement about the quality of the therapeutic alliance have been mixed, where some report a significant association between their ratings [21,22,24,25] and others do not [14,17,26]. Some studies however indicate that patients give higher ratings than the professional regarding the level of alliance [17,21,22,26,27] while others report no significant difference between patient and therapist scores [23].

The main body of studies have investigated the therapeutic relationship in the context of specific treatments, including cognitive-behavioral therapy (CBT) and cognitive remediation therapy [13-15,17,23,25]. Most studies have so far investigated samples of patients with a relative long duration of illness, where the quality of the working alliance may be influenced by negative outcomes or previous treatment failures. There are a very limited number of studies investigating the therapeutic alliance in early psychosis. One study reported that the therapeutic alliance mediated the apparent age effect on outcome in a study of CBT in first-episode patients, but without reporting descriptive data on the actual alliance itself [28]. One study of working alliance in early psychosis examined first-episode patients engaged in group therapy only [25], thus limiting the generalizability of their results to other treatment settings. A previous study from the latter group is, to our knowledge, the only one that reports on correlates to the level of therapeutic alliance in first-episode patients engaging in individual treatment relations and finds that friendship, leisure activates, quality of life, levels of insight, and medication side effects predicted 22% of the variance in the levels of patients’ working alliance [29] where the latter three were negatively associated with better alliances. The study however did not report on therapist ratings of the alliance and the association between patients’ and therapists’ reports. Omitting therapist scores leaves out valuable information concerning to what extent patients and therapists share opinion on the nature of their therapeutic relation. This is of importance as patient and therapist agreement on the alliance is associated with more positive treatment outcomes [19].

This is a cross-sectional study of a narrowly defined sample, conducted in a naturalistic treatment setting, with limited availability of previous research to guide the study design and formulation of clear-cut hypotheses. Thus, we chose an explorative approach to design and aim formulation, informed by a combination of available previous research, theoretical consideration, and available resources.

The purpose of the study is thus to explore the characteristics of the working alliance in patients with recent-onset schizophrenia spectrum disorders, with the aim of answering the following questions: Does patient clinical and cognitive characteristics influence patients’ and therapists’ reports of working alliance in recent-onset schizophrenia spectrum disorders? To what extent is there an association between patients’ and therapists’ ratings of the therapeutic alliance in this sample?

On the basis of previous research and theoretical consideration, we hypothesized that higher levels of psychotic symptoms would be associated with lower levels of both patient and therapist ratings of the working alliance. We also hypothesized that poorer patient insight would be associated with lower levels of both patient and therapist ratings of the working alliance. With regard to cognitive factors, we hypothesized from previous research that poorer verbal memory would be associated with higher levels of patient working alliance ratings, but unrelated to therapist ratings. For other cognitive functions, we assumed the zero hypothesis that they would be unrelated to the working alliance, as no previous reports on this were available (available resources to this study did not include measures of visuo-spatial reasoning, preventing the replication of previous results on this).

Due to mixed results from previous reports, we assumed the zero hypothesis that patient and therapist ratings of the working alliance would be of equal levels and that they would not be associated with each other.

## Methods

### Design

Subjects were recruited from out- and inpatient services at the Division of Psychiatry, St. Olav’s Hospital, Trondheim University Hospital, Norway (catchment area of approximately 230,000 inhabitants over the age of 18) as a part of the Thematically Organized Psychosis (TOP) research study [7]. The study was cross-sectional, and patients were included consecutively in the period from December 2006 to November 2011. The study was approved by the Regional Committee for Medical Research Ethics and the Norwegian Data Inspectorate. Participants’ written informed consent was obtained according to the Declaration of Helsinki.

### Procedure

Clinical psychologists and psychiatrists were trained specifically in the use of the applied measures and completed the assessments. All diagnoses, symptom assessments, and scorings were then re-evaluated and discussed with the first author and consensus scorings applied. The first author had completed the comprehensive training program used by the TOP study (based on the program used at the Semel Institute for Neuroscience and Human Behavior at the University of California, Los Angeles). The TOP study’s program shows good diagnostic reliability both towards gold standard training videos and for blinded expert scorings of randomly selected case vignettes from the actual sample. The inter-rater reliability of PANSS subscales for the training program were in the range of 0.73–0.82 (intraclass coefficient) (see [30] and [31] for details).

Therapists were the patients’ primary treating psychiatrist or psychologist at the hospital. Working alliance self-report forms were filled in by patients and therapists separately the same week or as soon as possible after symptom assessments at inclusion (within the first year of current treatment; see inclusion criteria for details) and placed in closed envelopes. All diagnoses and symptom assessments were thus blind to working alliance scores. The nonpharmacological treatment provided at the hospital for schizophrenia spectrum disorders is routinely given within an eclectic framework of interpersonal cognitive-behavioral theories. The procedure is in accordance with previous reports suggesting that alliance ratings do not fluctuate through the early phase of treatment and that different treatment approaches are not associated with different levels of the therapeutic alliance [20]. Patients were normally attending therapeutic sessions with their therapist once a week.

### Subjects

The inclusion criteria were as follows: (1) age 18–65 years; (2) meeting the Diagnostic and Statistical Manual of Mental Disorders, Fourth Edition (DSM-IV) criteria for a schizophrenia spectrum psychosis, including schizophrenia, schizophreniform disorder, schizoaffective disorder, and delusional disorder; (3) having the capability of supplying written informed consent; and (4) language abilities to complete the neurocognitive test battery. Exclusion criteria for patients were as follows: a history of moderate/severe head injury, neurological disorders, or mental retardation (IQ less than 70), in addition to comorbid cluster A or B personality disorder according to DSM-IV (determined by clinical consensus based on the evaluation of the DSM-IV criteria). Patients were eligible for inclusion up to 2 years after first meeting the criteria for a schizophrenia spectrum psychosis as defined above and up to 1 year after their first meeting with their current therapist. These criteria were chosen on the basis of research literature on the field of psychosis indicating the importance of the first years of illness in relation to treatment and outcome [2,3]. Also, previous research suggests that both patients and therapists give stable ratings of the therapeutic alliance during the early phase of treatment [20]. A cross-sectional assessment of the therapeutic alliance within the first year of the therapeutic relationship was thus considered acceptable as an early alliance measure. In addition, these were both considered appropriate criteria in order to obtain an adequate-sized sample within the study period. Of those who consented, four were found ineligible, due to longer duration of illness than accepted by inclusion criteria (two) and because of inadequate language abilities for completing the neurocognitive test battery (two), one were diagnosed with bipolar disorder, one withdrew the consent, and one dropped out without actively withdrawing consent. The study thus includes 42 subjects: 36 (85.7%) with schizophrenia, 3 (7.1%) with schizoaffective disorder, and 3 (7.1%) with delusional disorder; 28 (66.7%) men and 14 (33.3%) women; 28 (66.7%) inpatients and 14 (33.3%) outpatients at inclusion. Thirty-three (78.6%) were prescribed antipsychotic medication, four (9.5%) were prescribed mood stabilizers, one (2.4%) had antidepressive medication only, and eight (19%) did not use any psychotropic drugs. The mean duration of current antipsychotic treatment was 4.8 (±6.7) months (min 0.25, max 36). Eight (19%) met the DSM-IV criteria for substance abuse, six (14.3%) for substance dependence, one for alcohol abuse (2.4%), and five (11.9%) for alcohol dependence. On average, subjects had 2.8 (±3.6) psychiatric hospitalizations (min 0, max 18), a mean age of 27.5 (±5.6) years (min 20, max 51), and 11.8 (±1.9) years of education (min 9, max 17).

### Measurements

#### Clinical assessment

Diagnoses were established by the use of the Structured Clinical Interview for Diagnostic and Statistical Manual of Mental Disorders, Fourth Version [32]. Symptoms were assessed with the PANSS [33] after interview with the Structural Clinical Interview for the Positive and Negative Symptom Scale [34]. For the present study, we used a five-factor solution for scoring scale components derived from a first-episode sample [35]: The *PANSS positive factor* consists of the PANSS items P1 (delusions), P3 (hallucinatory behavior), P5 (grandiosity), P6 (suspiciousness/persecution), G9 (unusual thought content), and G12 (lack of judgment and insight); the *PANSS disorganized factor* consists of the PANSS items P2 (conceptual disorganization), N5 (difficulty in abstract thinking), N7 (stereotyped thinking), G5 (mannerisms and posturing), G10 (disorientation), G11 (poor attention), and G15 (preoccupation); the *PANSS negative factor* consists of the PANSS items N1 (blunted affect), N2 (emotional withdrawal), N3 (poor rapport), N4 (passive/apathetic social withdrawal), N6 (lack of spontaneity and flow of conversation), G7 (motor retardation), and G16 (active social avoidance); the *PANSS depressive/anxious factor* consists of the PANSS items G1 (somatic concern), G2 (anxiety), G3 (guilt feelings), G4 (tension), and G6 (depression); and the *PANSS excitative factor* consists of the PANSS items P4 (excitement), P7 (hostility), G8 (uncooperativeness), and G14 (poor impulse control). Insight was measured with the PANSS item G12 (‘failure to recognize past or present psychiatric illness or symptoms, denial of need for psychiatric hospitalization or treatment, decisions characterized by poor anticipation of consequences, and unrealistic short-term and long-range planning’).

#### Neurocognitive measures

Neurocognitive functioning was assessed by the use of a standardized neuropsychological test battery comprised of tests chosen for their relevance to schizophrenia spectrum disorders. The tests were administered in a fixed order by trained clinical psychologists. The tests consisted of the following: *Current general intellectual ability* was estimated by the use of four subtests from the Wechsler Adult Intelligence Scale (WAIS-III): Similarities, Vocabulary, Block design and Matrixes [36]. *Verbal memory* was measured by the California Verbal Learning Test II (CVLT-II) [37]. The test requires the patient to verbally recall (in five consecutive immediate recall trials) from a list of 16 words read by the test administrator as many words as possible. Verbal recall of the same 16 words was assessed with the 30-min delayed recall. *Executive functions* were measured by the Wisconsin Card Sorting Test Computer Version 2 - Research Edition (WCST: CV2): perseverative responses and perseverative errors [38]. *Attention* was measured by Conners’ Continuous Performance Test II (CPT-II): omissions and commissions [39].

#### Working Alliance Inventory - Short Form - client and therapist forms

Therapeutic alliance was assessed with the Working Alliance Inventory - Short Form (WAI-S) [40], a 12-item short version of the Working Alliance Inventory (WAI) [41]. The inventory is based on Bordin’s [42] formulation of the working alliance consisting of the therapeutic bond between client and therapist as well as their agreement on therapeutic goals and the tasks attended to in treatment. The WAI is a statement-based self-report measure with corresponding therapist and patient versions. Each statement is answered on a seven-point scale by indicating to what degree the statement is true (1 = never, 2 = rarely, 3 = now and then 4 = sometimes, 5 = often, 6 = very often, 7 = always). The WAI-S consists of 12 items of which two statements (items 4 and 10) are formulated as negations and scores reversed before computing total scores. Reliability analyses confirmed high internal consistency for WAI-S therapist and patient total scores and therapist and patient subscores for tasks, goals and bond, with Cronbach’s alpha ranging from .69 to .89.

### Statistical analyses

Statistical analyses were performed by the use of the Statistical Package for the Social Sciences, version 19 [43]. Because not all relevant variables were normally distributed, we used nonparametric tests in the bivariate analyses (Spearman’s correlations and Mann–Whitney *U* test for two independent samples). Tests were two-tailed and had a preset level of significance of 0.05. PANSS item G12 was used to measure insight in our analyses. As this item is also included in the PANSS positive scale, additional bivariate analyses with item G12 excluded from the PANSS positive scale were conducted. Results were not altered by the omission of item G12 from the PANSS positive scale. Hence, the original scale was kept unaltered. Hierarchical linear regression analyses (method: enter) with patients’ and therapists’ total WAI-S scores as dependent variables were performed to assess the individual contribution from variables with a significant bivariate association to the WAI-S total and subscores, including possibly confounding demographic variables, such as age and education. Model fit was evaluated through the visual examination of residual plots. Due to administrative error, one PANSS item G12 (measuring insight) as well as one patient and two therapist working alliance assessment forms were missing. Also, due to individual difficulties in completing all tests, CVLT-II and WCST were not administered for one patient and CPT-II for two patients. All missing scores were replaced by mean scores for the total sample in our analyses.

## Results

There were no gender differences or differences between inpatients and outpatients in neither patients’ nor therapists’ WAI-S total or subscores. Patients with longer education reported higher levels of WAI-S total and treatment task and goal scores. Older patients reported higher levels of the treatment task score (Table 1). There were no associations between demographic patient characteristics and therapists’ scores (Table 1).

**Table 1:** Spearman’s correlations (rho) between WAI-S scores and patient symptoms, cognition, insight, and demographic characteristics

	**WAI-S patient Spearman’s rho**				**WAI-S therapist Spearman’s rho**			
	**Total**	**Tasks**	**Goals**	**Bond**	**Total**	**Tasks**	**Goals**	**Bond**
PANSS								
Positive	−.279	−.262	−.358*	−.128	−.137	−.064	−.129	−.162
Disorganized	−.263	−.290	−.193	−.177	−.143	−.107	−.186	−.053
Negative	−.141	−.290	−.193	−.177	−.338*	−.350*	−.188	−.203
Depressive/anxious	−.059	−.124	−.126	−.013	.041	.096	.126	−.119
Excitative	−.337*	−.496**	−.147	−.306*	−.225	−.127	.016	−.398**
Insight								
PANSS item G12	−.089	−.040	.016	−.159	−.394**	−.349*	−.417**	−.259
Age	.244	.323*	.104	.242	−.020	−.091	−.130	.175
Education								
Total years	.414**	.394*	.323*	.303	.021	−.025	−.101	.174
Neurocognition								
WAIS-III								
Vocabulary	.010	.014	.190	−.102	−.107	−.119	−.082	−.139
Similarities	−.046	−.135	.194	−.089	−.159	−.167	−.029	−.279
Block design	.017	.000	.210	−.033	−.052	−.108	−.105	.021
Matrixes	.069	−.030	.175	.040	−.143	−.157	−.052	−.211
CVLT-II								
Long delay free recall	.020	−.002	.070	−.035	−.024	−.069	.017	−.032
WCST								
Perseverative responses	.013	−.088	.015	.109	.191	.078	.248	.111
Perseverative errors	−.087	−.194	−.083	.024	.155	.077	.225	.054
CPT-II								
Omissions	.035	.075	−.182	.138	−.139	−.102	−.078	−.056
Commissions	−.217	−.160	−.295	−.103	−.017	−.075	.011	.007

Significant correlations in italic numbers. WAI-S, Working Alliance Inventory - Short Form; PANSS, Positive and Negative Syndrome Scale (positive = P1, P3, P5, P6, G9, G12; disorganized = P2, N5, N7, G5, G10, G11, G15; negative = N1, N2, N3, N4, N6, G7, G16; depressive/anxious = G1, G2, G3, G4, G6; Excitative = P4, P7, G8, G14); WAIS-III, Wechsler Adult Intelligence Scale; CVLT-II, California Verbal Learning Test II; WCST, Wisconsin Card Sorting Test; CPT-II, Conners’ Continuous Performance Test II. *p < .05; **p < .01.

Higher scores (i.e., higher symptom loads) for the PANSS Excitative factor was associated with lower patient WAI-S total scores, in addition to lower levels of several subscores. Higher scores for the PANSS Positive factor was associated with lower patient WAI-S goal subscores (Table 1). High scores for the PANSS Negative factor was also associated with lower levels of therapists’ WAI-S total scores, in addition to therapists’ WAI-S task subscores (Table 1). The level of PANSS insight (G12) was not associated with patients’ WAI-S scores but negatively associated with WAI-S therapist total scores in addition to several subscores (Table 1). There were no association between any of the neurocognitive scores and patients’ or therapists’ WAI-S ratings (Table 1).

Multivariate linear regression analyses indicated that the best model explaining the variation in patients’ WAI-S total score was the patient’s age and level of excitative symptoms, which explained 23% of the observed variance (*R*^2^ = 0.19). For therapist WAI-S total scores, the regression analysis indicated that only insight (PANSS item G12) had a statistically significant influence and could explain 17% of the observed variance (*R*^2^ = 0.14) (Table 2).

**Table 2:** Multivariate linear regression analyses of patients’ and therapists’ WAI-S total scores

	**Model summary**				**Partial effects**				
	**Adjusted *R***^**2**^	***R***^**2**^	***F***	**Significance**	***β***	***t***	***p***	**95% CI**	
								**Lower**	**Upper**
Patient WAI-S total score	0.19	0.23	5.7	0.007					
Age					0.36	2.55	0.015	0.15	1.29
+ PANSS excitative					−0.28	−2.00	0.052	−2.85	0.01
Therapist WAI-S total score	0.14	0.17	7.9	0.008					
Insight (PANSS G12)					−0.41	−2.81	0.008	−4.05	−0.66

WAI-S, Working Alliance Inventory - Short Form; PANSS, Positive and Negative Syndrome Scale.

There were no significant differences between patient and therapist WAI-S total scores (ES 0.09), but with marginally larger variation in patient ratings (Table 3). Patients’ and therapists’ WAI-S total scores were statistically significantly associated (Table 4) and also had moderate degrees of association on the subscore level (Table 4).

**Table 3:** Descriptive statistics for psychotic symptoms, neurocognition, and working alliance

	**Mean ± SD**	**Min**	**Max**	**Median**
PANSS				
Total score	66.9 ± 15.6	49	115	63.0
Positive (P1, P3, P5, P6, G9, G12)	17.0 ± 5.6	6	33	16.0
Disorganized (P2, N5, N7, G5, G10, G11, G15)	13.1 ± 4.3	7	24	12.0
Negative (N1, N2, N3, N4, N6, G7, G16)	15.4 ± 6.1	7	28	15.0
Depressive/anxious (G1, G2, G3, G4, G6)	13.4 ± 4.5	5	23	13.5
Excitative (P4, P7, G8, G14)	5.9 ± 2.2	4	13	6.0
Item G12 insight	2.3 ± 1.3	1	6	2.2
WAI-S patient				
Total score	61.6 ± 11.1	31	79	64.0
Task score	20.4 ± 4.1	8	28	20.2
Goal score	20.9 ± 3.8	12	27	21.5
Bond score	20.3 ± 4.9	7	28	20.7
WAI-S therapist				
Total score	62.4 ± 7.7	38	80	62.2
Task score	20.4 ± 3.4	12	28	20.2
Goal score	20.6 ± 3.0	10	26	20.8
Bond score	21.5 ± 2.6	15	26	21.5
WAIS-III (scaled scores)				
Vocabulary	9.71 ± 2.6	6	18	9.5
Similarities	9.74 ± 3.3	5	18	9.0
Block design	11.43 ± 3.6	5	19	11.0
Matrixes	11.48 ± 3.3	4	18	12.0
CVLT-II (*z* score)				
Long delay free recall	−0.42 ± 1.5	−4	1.5	−0.2
WCST (T scores)				
Perseverative responses	45.49 ± 7.9	21	59	45.2
Perseverative errors	44.73 ± 8.2	20	63	45.0
CPT-II (*T* scores)				
Omissions	54.54 ± 12.8	40.9	101.1	49.9
Commissions	59.44 ± 11.1	38.1	79.9	61.6

WAI-S, Working Alliance Inventory - Short Form; PANSS, Positive and Negative Syndrome Scale; SD, standard deviation; WAIS-III, Wechsler Adult Intelligence Scale III; CVLT-II, California Verbal Learning Test II; WCST, Wisconsin Card Sorting Test; CPT-II, Conners’ Continuous Performance Test II.

**Table 4:** **Spearman**’**s correlations (rho) between WAI-S patient and WAI-S therapist total and subscores**

		**WAI-S patient Spearman**’**s rho**			
		**Total**	**Tasks**	**Goals**	**Bond**
WAI-S therapist Spearman’s rho	Total	0.394**	0.354*	0.342*	0.321*
	Tasks	0.372*	0.305*	0.262	0.357*
	Goals	0.237	0.171	0.239	0.162
	Bond	0.409**	0.426**	0.365*	0.298

Significant correlations in italic numbers. WAI-S, Working Alliance Inventory - Short Form. *p < .05; **p < .01.

Due to the observation that WAI-S patient and therapist total scores were moderately associated in the bivariate analyses, three separate hierarchical regression analyses were conducted to clarify if their association could be due to a statistical confounding effect of any of the variables previously identified as significant predictors of the two WAI-S total scores (i.e., patient’s age, PANSS Excitative factor or PANSS Insight (G12)). The relevant variables were entered in separate blocks with WAI-S total therapist score at the last step (block 2) and WAI-S total patient score as the dependent variable. The analyses indicated that neither acted as confounding variables on the association between WAI-S patient and therapist total scores.

## Discussion

The main finding was that that the patient’s age and level of excitative symptoms were the primary predictors of patients’ ratings of working alliance, while the level of insight was the strongest predictor of therapists’ ratings. There was no indication of any associations between neurocognitive factors and working alliance. This has been suggested, but not consistently found, in previous studies from more chronic groups [12,13,15,17,19,20]. Overall, there was a moderate degree of shared opinion between patient and therapist ratings of the quality of the working alliance, which was not influenced by the previously identified predictors of patients’ and therapists’ alliance ratings. This may be an expression of the unique relational aspect of the working alliance or pertain to other mediating variables that were possibly not included in the current study.

The association between excitative symptoms and patients’ experience of global working alliance with therapists seems reasonable from a clinical perspective, as excitement, hostility, uncooperativeness, and poor impulse control are behaviors contingent with negative relational experiences. Such an association has also been described in older groups of patients with schizophrenia spectrum disorders [19] although not consistently [12]. The multivariate regression analyses indicated that the bivariate association between total years of education and patient total alliance scores was the result of an association between age and education, as only age remained a significant variable in the regression model predicting patient working alliance total scores. This statistical association may be due to older patients having had the opportunity to complete more years of education, younger patients having an earlier onset of illness and thus earlier termination of education, or some other relation between age and education in this sample that cannot be identified from our analyses. Nevertheless, the finding is in line with findings from older and more chronic patients, who do not indicate any relationships between education and alliance [13].

The current study does not reproduce previous findings from more chronic patient samples showing statistically significant associations between working alliance and positive (psychotic) symptoms, negative symptoms, and levels of insight [13,15,17]. When contrasted with the abovementioned previous research, the present findings suggest that there may be a qualitatively different pattern of associations between patient characteristics, insight, and working alliance in the early treatment of recent-onset schizophrenia spectrum disorders relative to patients with longer duration of their illness. Insight (i.e., the quality of patient awareness and understanding of own psychiatric condition and degree of withdrawal) was the only factor influencing the therapists’ scores. Thus, therapists should be aware of how this may blur their perception of the quality of the therapeutic relation as the patient sees it and also of the effect of the lack of insight on his/her personal experience of the cooperation and bond with the patient in the early phases of treatment. Both aspects could possibly exert a secondary effect on therapist behaviors.

Looking at the WAI’s subscale level, patient and therapist ratings of their relational bond were associated with excitative symptoms only, suggesting that both parts’ experience of a poorer relational bond is specifically associated with the more unstable, hostile, and uncooperative symptoms. Still, patient and therapist ratings of the relational bond are not consistently associated with each other, suggesting that other factors also influence bond scores. Thus, the data support the notion that the therapeutic alliance is a complex phenomenon. Patient and therapist ratings of agreement on treatment tasks showed the same patterns of association as their working alliance total scores, perhaps illustrating the higher impact of these two subscales on the working alliance total score.

Patient ratings of less agreement on treatment goals were differentially associated with patients having shorter education and more positive psychotic symptoms, i.e., delusions, hallucinatory behavior, grandiosity, suspiciousness/persecution, unusual thought content, and lack of judgment and insight, in agreement with previous findings [13]. Therapist ratings of less agreement on treatment goals were solely associated with patients showing poorer judgment and insight. These results seem to mirror a well-known challenge in clinical work, when negotiating agreement on treatment goals with patients and there is little or no shared or common perception of reality: The delusional or hallucinating patient experiences the therapist as working towards different goals as himself or herself, and the therapist who struggles with establishing joint goals experiences the patient as presenting with lack of insight. One could argue that due to the lack of shared reality, differential patterns of factors that are associated with patient and therapist perspectives on the working alliance are to be expected in therapeutic work with psychotic patients.

In the present study, WAI-S total score levels and distributions for patients and therapists were fairly high and comparable to those reported in other studies of schizophrenia spectrum samples using the same scale [13,17,19]. There was a moderate association between patient and therapist global ratings of the working alliance, but with a higher concordance in alliance total scores between patients and therapists than in previous studies [12,13,15,18,19]. Multivariate linear regression analyses indicated that the association between patient and therapist working alliance total scores were not confounded by other predictors of the total scores. The higher degree of shared opinion on the working alliance found in this study compared to previous studies possibly reflects a better quality of the therapeutic alliance with patients with recent-onset schizophrenia spectrum disorders as compared to older patients with longer duration or more chronic courses of illness. If so, this adds weight to the importance of the early psychosocial treatment for patients with these disorders.

On the subscale level, the differential characteristics of patient and therapist perceptions of the alliance were reflected in that both goal scores and bond scores were unrelated, while task scores were moderately associated. The strongest subscale associations were between patient experience of more agreement on treatment tasks and goals and therapist experience of a stronger relational bond. At close to the same level was the association between patient experience of a stronger relational bond and therapist experience of more agreement on treatment tasks. Thus, the results from the bivariate analyses of concordance between patient and therapist perceptions of the working alliance indicate that patients and therapists may perceive interpersonal aspects of treatment differently or be differentially affected by events experienced within the cooperative framework of the therapeutic alliance.

We hypothesized that higher levels of psychotic symptoms would be associated with lower levels of both patient and therapist ratings of the working alliance and that poorer patient insight would be associated with lower levels of both patient and therapist ratings of the working alliance. These hypotheses were thus partly supported, in that patient working alliance scores were predicted by excitative symptoms and therapist working alliance scores were predicted by patient insight. With regard to cognitive factors, the hypothesis that poorer verbal memory would be associated with higher levels of patient working alliance ratings was not supported, but the zero hypothesis that other neurocognitive factors would be unrelated to working alliance was confirmed. Concerning patient and therapist agreement on the working alliance, the zero hypothesis that patient and therapist working alliance scores would be equal in levels was confirmed but had to be rejected with regard to their bivariate association.

### Limitations

The study sample was of moderate size which may limit generalizability. Participating patients were not part of a standardized treatment program but assessed in a naturalistic treatment setting at the hospital psychiatric department. This lessened the possibility to standardize the timing of assessments in detail and theoretically increased the number of possible confounding variables. The inclusion of subjects in this study was based on informed consent. Thus, it is possible that there has been an implicit selection of adherent patients. However, some patients expressed motives for participating that were not necessarily related to adherence to treatment, such as wanting to contribute for the benefit of other patients, wanting to give their opinion on aspects of the provided healthcare that they found insufficient, or wanting a more in-depth neurocognitive examination than provided in ordinary treatment. From a theoretical point of view, one could suspect that nonconsenting patients would experience poorer quality of therapeutic alliances and more disturbing levels of psychotic symptoms than would patients who gave consent. Still, due to the design of the study, we do not know if they did in fact exhibit substantially different characteristics on the variables of interest than those who consented.

## Conclusion

Patients’ and therapists’ qualitative experience of the working alliance is associated with specific but different patient characteristics in recent-onset schizophrenia spectrum disorders. These may be phase-specific characteristics associated with the quality of the early alliance. Patients in the early phase share opinion with their therapists on the quality of the working alliance to a higher degree than reported in previous studies of patients in the later phases of illness.

### Implications

These results provide new knowledge about the importance of patients’ individual profiles of symptoms in recent-onset schizophrenia spectrum disorders, with regard to their association with the quality of the working alliance. This is of importance in order to inform clinicians’ alliance enhancing efforts in the early course of treatment, as it may contribute to the prevention of drop-out and to optimize compliance and outcome. This study has shown that good-quality working alliances can be achieved in recent-onset schizophrenia spectrum disorders and that a fair degree of agreement on this alliance can be achieved between patient and therapist. Taken together, these findings state the importance of utilizing the opportunity for early treatment gains and may improve therapist interventions when providing psychosocial treatment to patients with recent-onset schizophrenia spectrum disorders.

## Competing interests

The authors declare that they have no competing interests.

## Authors’ contributions

RJ designed and conducted the study, analyzed and interpreted the data, and wrote the first and final drafts of the manuscript. KAH has made substantial contribution to the conception and design of the study, analysis and interpretation of the data, and critical revision of the manuscript for all intellectual content. IM has made substantial contribution to the design of both this study and the TOP study that it originates from. She contributed substantially to the analysis and interpretation of the data and critical revision of the manuscript for all intellectual content. VCI has contributed substantially to the analysis and interpretation of the data and critical revision of the manuscript for all intellectual content. All authors have read and approved the final manuscript.
